# Editorial: Vaccines against parasitic infections in domestic animals

**DOI:** 10.3389/fvets.2023.1144700

**Published:** 2023-02-16

**Authors:** Luiz Daniel de Barros, Camila Koutsodontis Cerqueira-Cézar

**Affiliations:** ^1^Laboratory of Animal Protozoology, Department of Preventive Veterinary Medicine, Universidade Estadual de Londrina, Londrina, Brazil; ^2^Department of Veterinary Hygiene and Public Health, Universidade Estadual Paulista “Júlio de Mesquita Filho” (UNESP), Botucatu, Brazil

**Keywords:** vaccination, parasites, arthropods, protozoa, helminths

Parasitic infections are responsible for relevant diseases in domestic animals, including pets and livestock. These infections caused by protozoans, arthropods, and helminths, may lead to huge economic losses, together with zoonotic potential from several of them. It is estimated that more than 75% of human diseases have an animal origin, and parasites contribute to this high prevalence (1). Currently, parasite control is done mainly by chemical agents, which can damage the environment, leave drug residues in animal products, and contribute to the emergence of resistant parasites. Thus, scientists worldwide have invested efforts in developing vaccines to prevent parasitic infections and reduce the adverse effects of chemotherapy.

A safe and successful vaccine should induce a long-lasting immune response but also provide protection for the host against other existent strains of the target pathogen (2). The development of an effective vaccine regarding parasitic infections is challenging, as parasites have complex life cycles and can usually evade their host's immune system, using mechanisms that are not fully understood yet (3). The creation of effective vaccines for parasitic diseases is of paramount importance as it would decrease the prevalence of the diseases in human and animal populations.

Owing to the importance of parasitic infection in animals, this Research Topic on Vaccines Against Parasitic Infections in Domestic Animals focused on new advances in vaccines against arthropods, protozoa, and helminths of veterinary interest. In total, seven papers, including six original research articles and one review, from 56 authors from Bangladesh, China, Japan, Spain, Switzerland, Thailand, the United Kingdom, and the United States were included in this topic. Figure 1 shows the nationalities of all authors that contributed to this Research Topic according to the parasite species.

**Figure 1:**
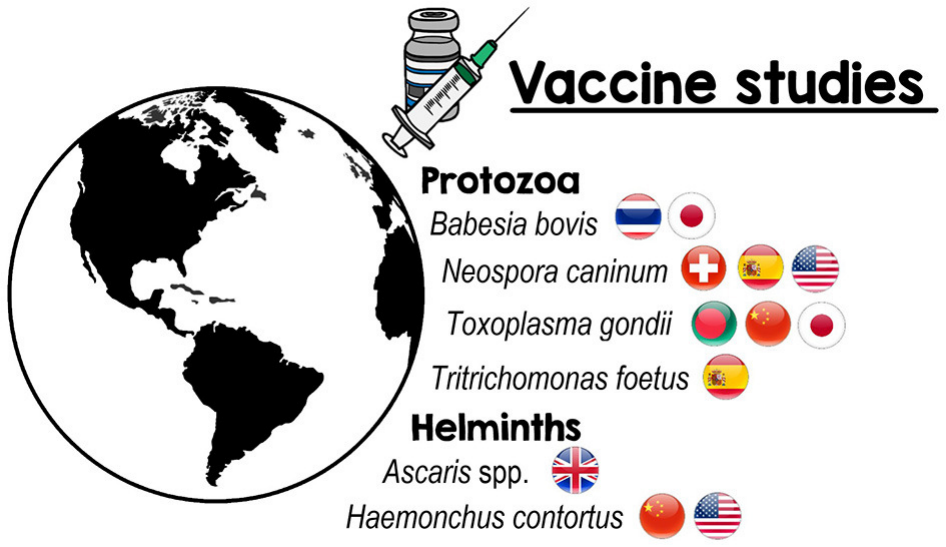
Countries of all authors that contributed to the Research Topic: vaccines against parasitic infections in domestic animals according to the parasites.

The review by Hasan and Nishikawa introduced the recent advances in vaccine development against *Toxoplasma gondii* and the immunological aspects of the disease in sheep and goats. The authors listed the recent studies based on live, tachyzoites, DNA, vector, and nanoparticles vaccines against toxoplasmosis in small ruminants, highlighting the importance of vaccination for the control of congenital infection and, consequently, reproductive disorders as well as acute and chronic disease for production of safe meat for human consumption.

Among the original research articles, four were related to vaccines against protozoan parasites. Using a vaccination-drug treatment approach in mice, Imhof et al. demonstrated a higher efficacy of the Listeria monocytogenes vaccine expressing the tachyzoite surface antigen NcSAG1 (Lm3Dx_SAG1) of Neospora caninum plus bumped kinase inhibitor BKI-1748 treatment than only vaccination or drug treatment. These results indicated that vaccination-drug treatment could be a potential strategy against *Neospora* infection. The study performed by Rittipornlertrak et al. evaluated the immune response of cattle immunized with the recombinant structural ectodomains I and II of the apical membrane antigen 1 (AMA-1) of *Babesia bovis*. The authors observed that vaccinated cattle developed a strong Th1 immune response, with higher titers of IgG antibodies and eliciting CD4^+^ and CD8^+^ T cells producing interferon-gamma (IFN-γ) and tumor necrosis factor alfa (TNF-α). Based on these findings, AMA-1 was considered a vaccine candidate against bovine babesiosis. The evaluation of a new vaccine against *Tritrichomonas foetus* in cattle, called Trichobovis, was conducted by Ortega-Mora et al.. Based on a vaccine composed of *T. foetus* trophozoites plus Quil-A as an adjuvant, the authors observed that vaccinated cows cleared the infection before the control group and developed IgG and genital IgA similar to the commercial vaccine available. Furthermore, the reproductive performance of vaccinated cows improved in a field experiment, demonstrating the beneficial effects of this new vaccine. The evaluation of a vaccine against chronic *Toxoplasma gondii* infection in mice was published by Tian, Yang et al.. Using a vaccine based on *T. gondii* bradyzoite-formation deficient 1 (TgBFD1), it was observed that vaccinated mice produced high levels of IgG, IgG1, IgG2, and the cytokines IFN-γ and interleukin 10. Although the vaccine was ineffective against acute infection, it led to fewer cysts in the brains of vaccinated mice. These results suggest that this vaccine could be a candidate for controlling toxoplasmosis in animals.

Regarding the original research articles describing vaccines against helminths, two studies were published. In the first one, Tian, Lu et al. evaluated the efficacy of active and passive immunization of crossbred female goats with recombinant excretory-secretory products of *Haemonchus contortus* Y75B8A.8 (Hc8). It was observed that both immunization processes reduced cumulative fecal egg counts and worm burden. Besides, the active immunization produced higher levels of IgG anti-rHc8 than in non-immunized goats leading to stronger protection against *H. contortus* infection. Based on these results, the rHc8 antigens could be considered as possible vaccine candidates. The second article, by Evangelista et al., aimed to identify a potential vaccine against *Ascaris* infection using the reverse vaccinology technique. Four transmembrane proteins were identified to contain epitopes with high affinity to T and B-cells, evidencing potential antigens to be used in future studies of vaccines against *Ascaris* infection in animals and humans.

In conclusion, the Research Topic Vaccines Against Parasitic Infections in Domestic Animals contributed to introducing new vaccine candidates and strategies against parasitic infections in animals worldwide.

## Author contributions

LdB and CKCC were Topic Editors of the Research Topic and wrote and revised the editorial article. All authors contributed to the article and approved the final version.

## References

[1] Jones KE Patel NG Levy MA Storeygard A Balk D Gittleman JL. Global trends in emerging infectious diseases. Nature. (2008) 451:990–3. 10.1038/nature06536 18288193 PMC5960580

[2] Ivory C Chadee K. DNA vaccines: Designing strategies against parasitic infections. Genet Vaccines Ther. (2004) 2:1–8. 10.1186/1479-0556-2-17 15579202 PMC544584

[3] Zawawi A Else KJ. Soil-transmitted helminth vaccines: are we getting closer? Front Immunol. (2020) 11:576748. 10.3389/fimmu.2020.576748 33133094 PMC7565266

